# Research on Spatiotemporal Differentiation and Influence Mechanism of Urban Resilience in China Based on MGWR Model

**DOI:** 10.3390/ijerph18031056

**Published:** 2021-01-25

**Authors:** Yu Chen, Mengke Zhu, Qian Zhou, Yurong Qiao

**Affiliations:** 1 School of Economics and Management, Zhengzhou University of Light Industry, Science Avenue 136, Zheng Zhou 450000, China; 2012030@zzuli.edu.cn (Y.C.); 13939045355@163.com (M.Z.); qiaoyurong9@163.com (Y.Q.); 2 Economics School, Zhongnan University of Economics and Law, Nanhu Avenue 182, Wuhan 430073, China

**Keywords:** urban resilience, spatiotemporal differentiation, MGWR, spatial scale, factors of urban resilience

## Abstract

Urban resilience in the context of COVID-19 epidemic refers to the ability of an urban system to resist, absorb, adapt and recover from danger in time to hedge its impact when confronted with external shocks such as epidemic, which is also a capability that must be strengthened for urban development in the context of normal epidemic. Based on the multi-dimensional perspective, entropy method and exploratory spatial data analysis (ESDA) are used to analyze the spatiotemporal evolution characteristics of urban resilience of 281 cities of China from 2011 to 2018, and MGWR model is used to discuss the driving factors affecting the development of urban resilience. It is found that: (1) The urban resilience and sub-resilience show a continuous decline in time, with no obvious sign of convergence, while the spatial agglomeration effect shows an increasing trend year by year. (2) The spatial heterogeneity of urban resilience is significant, with obvious distribution characteristics of “high in east and low in west”. Urban resilience in the east, the central and the west are quite different in terms of development structure and spatial correlation. The eastern region is dominated by the “three-core driving mode”, and the urban resilience shows a significant positive spatial correlation; the central area is a “rectangular structure”, which is also spatially positively correlated; The western region is a “pyramid structure” with significant negative spatial correlation. (3) The spatial heterogeneity of the driving factors is significant, and they have different impact scales on the urban resilience development. The market capacity is the largest impact intensity, while the infrastructure investment is the least impact intensity. On this basis, this paper explores the ways to improve urban resilience in China from different aspects, such as market, technology, finance and government.

## 1. Introduction

Urban resilience is the ability of a city to recover in the face of various disasters. Covid-19 is one of the most serious “black swan” outbreaks in global public health security in recent years. The outbreak, which has brought many cities of China and around the world to a standstill, has severely tested the ability of cities to repair themselves in the face of acute shocks and long-term stress. Faced with the rebound of the epidemic in many countries in Europe and the US, China has taken a series of commendable measures to combat the epidemic in the early stage of the outbreak. However, it is undeniable that the shortage of public health resources and the lagging early warning mechanism of the medical and health system have also been exposed one by one. In fact, this is already the sixth “public health emergency of international concern” (PHEIC) declared by the World Health Organization in 15 years, and public health events have become one of the great threats to contemporary urban development. However, with the increasingly complex urban operation system, for China in the critical period of structural adjustment and social transformation, the threats may also come from a series of risks and challenges such as periodic economic crisis, global climate change, and urban terrorist attacks. Faced with the constraints of many uncertain factors, how to ensure the stable survival and sustainable development of cities has become the focus of attention of governments and scholars all over the world [1,2]. In essence, the difference in the ability of cities to cope with risks is caused by the urban resilience of different cities [3]. As the ability of urban system to adapt to uncertainty, urban resilience can not only break the sustainability paradox from the perspective of imbalance [4], but also help urban system to digest and absorb external interference to the greatest extent, so as to maintain its original features and key functions [5]. For China, which is undergoing continuous industrialization and rapid urbanization, it undoubtedly provides us with a new research perspective, replacing “short-term pain relief” with “long-term pain treatment” [6], and maintaining urban operation in a more systematic and long-term way under the normal situation of the epidemic. Therefore, scientific measurement of the level and spatial evolution of China’s urban resilience and in-depth analysis of the spatial differentiation pattern of its driving factors will help speed up the clarification of the current development status of China’s urban resilience and point out the direction for the healthy development of cities in the new era.

This paper takes 281 cities in China from 2011 to 2018 as research objects. On the one hand, the evaluation system is constructed from the five aspects of economy, engineering, society, ecology and institution to investigate the spatiotemporal differentiation pattern of China’s urban resilience development. On the other hand, the driving factors are selected from the perspectives of government, technology, market, openness and finance. The multi-scale geographical weighted regression model (MGWR) is used to deeply analyze the effects of various variables on urban resilience at different impact scales, so as to provide reference basis for improving urban resilience in China.

### 1.1. Literature Review

#### 1.1.1. Origin and Evolution of Concepts

The term “resilience” first appeared in the field of mechanics to describe the ability of metals to recover after being deformed under external forces [3]. With the deepening of the research, the concept has undergone two thorough modifications and improvements from the initial engineering resilience to the ecological resilience and then to the evolutionary resilience. In a sense, engineering resilience is the closest to the concept of resilience commonly understood by people. It refers to the ability of the whole system to recover to the equilibrium or stable state before disturbance after being disturbed [7]. With the deepening understanding of system and environmental characteristics and their mechanism of action, ecologist Holling first applied the idea of resilience to ecology in 1973 and proposed ecological resilience. Ecological resilience breaks the limitation that engineering resilience holds that the system has a single equilibrium state, and emphasizes the sustainable development ability of the system [7]. On this basis, based on the adaptive cycle theory of Gunderson and Holling [8], the concept of evolutionary resilience was proposed, which pays more attention to the adaptability, learning and innovation ability of the system. Compared with engineering resilience and ecological resilience, evolutionary resilience is more convincing in theory, and it is also regarded as the reference benchmark for urban resilience research. Therefore, urban resilience can be regarded as the inheritance and redevelopment of the traditional resilience theory. It takes the urban ontology as the research object and emphasizes the ability of urban system to maintain its original main features, structure and key functions after encountering external harassment [5]. The research focuses on deconstructing the interactive logic relationship between modern city and its crisis risk, as well as discussing the systematic response measures taken by cities to deal with complex disturbances. At the same time, urban resilience is also regarded as an innovative approach to achieve sustainable development goals. Compared with the fail-safe concept emphasized in the early stage of sustainable development, urban resilience focuses on the integrity of the overall urban pattern and the sustainability of functional operation and is a safe-to-fail approach [9].

#### 1.1.2. Evaluation Method and System

The evaluation method and index system of urban resilience are the in-depth study of the theoretical framework of resilience, which is helpful to translate the theoretical analysis into urban construction. As a new research subject, the academic circle has not put forward a unified evaluation method for urban resilience. However, by definition, resilience can be measured by the amount of disturbance that the urban system can absorb if its original functional structure remains unchanged [10,11]. As the core of resilience measurement, disturbance threshold is difficult to be obtained directly and is mostly represented by multi-functional indexes. As interdisciplinary studies, many scholars try to build evaluation systems from different starting points, which are mainly divided into three categories: different dimensions, different characteristics, and different scales. Among them, urban resilience evaluation can construct index system from multiple dimensions and single dimension. From the perspective of multiple dimensions, scholars usually select comprehensive indicators from the aspects of economy, society, nature and infrastructure, etc. [12,13], while the single dimension usually takes a specific resilience [14] or natural disaster [15] as the object and constructs highly targeted evaluation indicators. The evaluation system based on different characteristics is mainly based on the seven characteristics of resilient cities proposed by Rockefeller Foundation. On this basis, some scholars have made a comprehensive classification description from individuals, organizations and localities [16]. However, it should be noted that there is still lack of quantitative models and unified scales to fit the relationship between different system characteristics and quantify the resilience of urban system. The evaluation scale of resilient cities can be divided into macro metropolitan area, medium single city and micro community. They are respectively represented by the Resilience Capacity Index (RCI) for metropolitan areas proposed by Berkeley Research Institute, the resilience city index system launched by Rockefeller Foundation of the United States, and the community resilience index which includes six elements of community infrastructure, security, environment, economy, system, society and population [17].

#### 1.1.3. Development Strategy and Path

Targeted promotion strategies are the ultimate goal of urban resilience evaluation research. The increasing research on urban resilience strategies reflects the rapid development trend of research from theoretical exploration to practical application. To be specific, urban resilience practice at the present stage has two main footholds. The first is based on the characteristics of resilience. Jack Ahern, a professor at the University of Massachusetts, United States, believes that resilient cities should have five characteristics: multi-function, redundancy and modularity, (biological and social) diversity, multi-scale networks and connectivity, and adaptive planning and design [18]. And on this basis, five strategies of urban resilience planning and design are proposed [9]. This emphasis on building urban resilience by some means, including providing sustainable ecosystem services in view of the limited space, the distributed system to provide the same functionality to strengthen risk dispersion ability, based on the diversity of the biological system reaction to form low impact development pattern, repeated set loop to maintain functional connectivity and the use of adaptive design decisions to the problem. The second is to refine to the practical level. According to the theory of urban resilience and relevant studies by scholars [19], the main ideas of urban resilience planning are as follows: Vulnerability analysis and evaluation, uncertainty planning for cities, strengthening the leading role of the government in urban governance and formulating specific implementation strategies, etc. In this way, we can formulate policies and measures based on future scenarios on the basis of clearly understanding the mechanism of vulnerability, so as to effectively coordinate the conflict of interests in the implementation process of resilient cities and avoid the occurrence of basic dysfunction and fault of service supply when the urban system is exposed to external threats.

#### 1.1.4. Summarize

In conclusion, the research on urban resilience has shifted from Bohr paradigm to Pasteur paradigm, and there are more practical scholars, making the research on resilience more timely and targeted, and overcoming the gap between “knowledge generation” and “knowledge transformation”. In terms of concept, although unified connotation has not been formed within the discipline, it does not affect the establishment of relevant theoretical framework and the development of research practice, but makes the research gradually extend from theoretical analysis to internal mechanism. In terms of evaluation, although no consensus has been reached on the quantitative method of urban resilience, it has made some initial achievements in the construction of resilience index system. The evaluation system is relatively comprehensive, covering different dimensions, characteristics and scales. Nevertheless, the index system that accords with China’s national conditions still needs to be improved. In terms of strategy, most of the existing studies provide guiding principles for decision-making and policy making of urban management. However, there is no in-depth study on the formation mechanism and influencing factors of urban resilience, and there is also a lack of discussion on spatial scale. Compared with the existing literature, the innovations and contributions of this paper are: (1) Evaluation index: Aiming at China’s current national conditions, this paper integrates traditional data and emerging data, and adds institutional dimension on the basis of four dimensions of economy, society, engineering and ecology to highlight the main role of government and non-governmental organizations in urban resilience development. (2) Research method: MGWR model is used to analyze the driving factors, which can effectively analyze the influencing factors from different spatial scales. Firstly, the city is placed in a macro background to consider the role of influencing factors on the overall dimension. Secondly, it is dynamic across multiple scales from regions to communities, even to families and individuals, presenting its spatial role. (3) Optimization path: In order to reflect the continuous relationship between promotion strategy and evaluation system, the mechanism of action of urban resilience in China is deeply explored, and the internal relationship between optimization path and evaluation system is further clarified according to its influencing factors.

## 2. Materials and Methods

### 2.1. Research Methods

#### 2.1.1. Entropy Method

The entropy method provides a basis for multi-dimensional comprehensive evaluation and is more objective, avoiding the influence of human factors in subjective assignment. Therefore, the entropy method is adopted in this paper to give weight to the urban resilience index system of China’s cities, and the urban resilience index, economic resilience index, engineering resilience index, social resilience index, ecological resilience index and institutional resilience index are calculated.

First, the range method is adopted to standardize the original data. According to the positive and negative of each index of urban resilience, the standardization is as follows:

(1) $$\mathrm{Positive}\;\mathrm{indicators}:\;x_{\mathrm{ij}}^{\prime }=\frac{x_{\mathrm{ij}}-\min\left(x_{\mathrm{ij}}\right)}{\max\left(x_{\mathrm{ij}}\right)-\min\left(x_{\mathrm{ij}}\right)}$$

(2) $$\mathrm{Negative}\;\mathrm{indicators}:\;x_{\mathrm{ij}}^{\prime }=\frac{\max(x_{\mathrm{ij})}-x_{\mathrm{ij}}}{\max\left(x_{\mathrm{ij}}\right)-\min\left(x_{\mathrm{ij}}\right)}$$

where: x_ij_ is the original value of index i in region j, $x_{\mathrm{ij}}^{\prime }$ is the standardized value, max(x_ij_) and min(x_ij_) are the maximum and minimum values of this index respectively.

Secondly, calculate the entropy value e_j_ of the index x_ij_:

(3) $$e_{j}=-k\overset{n}{\underset{i=1}{\sum }}p_{\mathrm{ij}}\times \ln\left(p_{\mathrm{ij}}\right)$$

In Equation (3), $P_{\mathrm{ij}}=\frac{x_{\mathrm{ij}}^{\prime }}{\sum_{i=1}^{n}x_{\mathrm{ij}}^{\prime }}$, k = 1/ln(n), n is the sample size, which satisfies e_j_ > 0.

Thirdly, calculate the urban resilience index and sub-resilience index S_i_ of each city:

(4) $$S_{i}=\overset{m}{\underset{j=1}{\sum }}w_{j}\times x_{\mathrm{ij}}$$

In Equation (4), $w_{j}=\frac{d_{j}}{\sum_{j=1}^{m}d_{j}}$ represents the weight of index j, where d_j_ = 1 − e_j_.

#### 2.1.2. Exploratory Spatial Data Analysis (ESDA)

ESDA is an ideal data-driven analysis method. It takes spatial association degree as the core and discovers spatial clustering and spatial anomaly by describing and visualizing spatial phenomena of objects, thus revealing the spatial interaction mechanism between research objects. It usually includes global spatial autocorrelation analysis and local spatial autocorrelation analysis.

##### Global Spatial Autocorrelation Analysis

Global Moran’s I index measures the overall trend of spatial correlation of unit attribute values in adjacent or adjacent regions in the whole study area.

(5) $$I=\frac{\sum_{i=1}^{n}\sum_{j=1}^{n}w_{\mathrm{ij}}\left(x_{i}-\;x\;¯\right)\left(x_{j}-\;x\;¯\right)}{S^{2}\sum_{i=1}^{n}\sum_{j=1}^{n}w_{\mathrm{ij}}}$$

In Equation (5), I is the global Moran’s I, n is the number of cities, x_i_ and x_j_ respectively represent the urban resilience of region i and j, and W_ij_ is the spatial weight matrix.

##### Local Spatial Autocorrelation Analysis

Local spatial autocorrelation mainly analyzes the different spatial association patterns that may exist at different spatial locations in order to find the spatial heterogeneity among data. In this paper, Local Moran’s I, also known as Local Indicator Spatial Association (LISA), is used to indicate the significance of spatial difference.

(6) $$\mathrm{LISA}=\frac{\left(x_{i}-\;x\;¯\right)}{S_{x}^{2}}\;\overset{n}{\underset{i=1}{\sum }}[w_{\mathrm{ij}}\left(x_{i}-\;x\;¯\right)]$$

In Equation (6): $S_{x}^{2}=\sum^{\;}{\left(x_{i}-\;x\;¯\right)}^{2}/n$. A positive LISA means a high value surrounded by a high value or a low value surrounded by a low value, namely, H-H or L-L. A negative LISA means that a low value is surrounded by a high value or a high value is surrounded by a low value, namely, H-L or L-H.

#### 2.1.3. Multi-Scale Geographically Weighted Regression (MGWR)

Compared with the traditional classical GWR model, MGWR makes its bandwidth specific by allowing variables to have different spatial smoothing levels, which solves the problem of limiting the optimal bandwidth of all variables to be the same in GWR. At the same time, the specific bandwidth of each variable can also be used as the index of the spatial scale of each spatial process, which makes the spatial process model generated by this multi-bandwidth method more real and useful [20]. Therefore, this paper uses the MGWR model to discuss the spatial differentiation and spatial scale differences of the driving factors of urban resilience in 281 cities of China from 2011 to 2018. The formula is as follows:

(7) $$y_{i}=\overset{k}{\underset{j=1}{\sum }}\beta_{\mathrm{bij}}\left(u_{i}\;,v_{i}\right)x_{\mathrm{ij}}+\varepsilon_{i}$$

In Equation (7), $\beta_{\mathrm{bij}}$ represents the regression coefficient of the local variable, bij represents the bandwidth used by the regression coefficient of the variable j, and $\left(u_{i}\;,v_{i}\right)$ represents the spatial coordinates of the sample point i, x_ij_ is the observed value of the variable j at the sample point i, and $\varepsilon_{i}$ is the random disturbance term.

### 2.2. Variable Selection

The “sustainability” of the world in the 21st century largely depends on the sustainability of cities, and urban construction planning is bound to transition from “quantitative accumulation” to “qualitative transformation”. In this historical tide, urban resilience has provided a new paradigm for urban development, enabling urban development strategies to respond to economic, social or environmental changes. In the future, urban resilience and urban development will be characterized by interactive coupling, and urban resilience can be enhanced through the regulation of urban construction. Before this, it is necessary to accelerate the development of an evaluation system to measure urban resilience. Urban resilience is a multi-dimensional interdisciplinary subject involving a wide range of areas [21]. Based on data availability, this paper classifies urban resilience into four dimensions: economic resilience, engineering resilience, social resilience and ecological resilience. In addition, different from previous urban security strategies, urban construction in the post-epidemic era should focus more on unknown and unpredictable risk shocks, and it is necessary to incorporate epidemic prevention and control planning into the territorial space planning system. In view of the important role played by the government and some non-governmental organizations in the epidemic prevention and control work, this paper adds the dimension of institutional resilience, so as to comprehensively analyze the development status of China’s urban resilience (Table 1).

Economic resilience emphasizes that the level of urban economic development should match the resilience to ensure the coordination and symbiosis among urban subsystems, so as to ensure the rapid resumption of production and construction in the event of external risks. Therefore, the indicators are selected from three aspects of economic strength, economic diversity and economic extroversion. Among them, the economic strength reflects the ability of economic security that a city can provide when dealing with external turbulence, and it is a direct manifestation of the city’s resilience. Economic diversity advocates the change of single economic structure and the adoption of diversified economy as a new development model, so as to maintain the diversity of urban economy and stratum. Economic extroversion requires cities to actively participate in the global economic division of labor. In the context of the global impact of the COVID-19 epidemic, China is at the forefront of the world economic recovery. All regions should make full use of their distinctive urban personalities and continue to consolidate their positions in the global industrial chain.

Engineering resilience is to evaluate whether the infrastructure and community construction are capable of rescue and emergency response in the event of occasional extreme events and crises in the city. It mainly selects indicators from the three criteria: urban evacuation ability, basic supply and drainage ability and external communication ability. The urban evacuation ability is mainly reflected in the traffic accessibility, which reflects the city’s ability to shelter and evacuate people when the crisis occurs. The basic supply and drainage ability reflects the service level of urban hardware facilities. Edwin Chadwick, in his Reporton the Sanitary Condition of the Labouring Population of Great Britain, pointed out that infectious, endemic and other diseases were caused, aggravated or spread by crowded, damp and dirty living conditions, and that the incidence of these diseases could be effectively reduced by means of drainage, electricity supply and cleaning [22]. External communication ability emphasizes that the response time should be compressed to the greatest extent when the epidemic or risk occurs, and the loss should be reduced by timely transmission of disaster information so as to effectively organize the urban social system.

Social resilience is the integration of factors such as demographic characteristics, organizational structure and human capital, which reflects the ability of cities to deal with the problems caused by social, political and environmental changes [23]. It mainly includes population adaptation ability, social security ability and risk response ability. Population adaptation ability reflects residents’ sense of belonging to the city and their ability to withstand crises. Social security ability and risk response ability emphasize that social resilience should attach importance to both the hard environment and the soft environment. The report of the 19th CPC National Congress clearly stated that to implement the healthy China strategy, cities must build a strong public health system under the normal situation of the epidemic, which is firstly reflected in the basic conditions of disease prevention and control, and secondly in the construction of the talent team for disease control.

Ecological resilience refers to the extent to which natural resources and ecosystems can resolve crisis changes before they are reorganized and formed into new structures. It can be measured in terms of environmental pressure, governance ability and service ability [24]. Environmental pressure is generally represented by the environmental emission intensity index of the industrial sector in the process of ecological restoration. Governance ability is a measure to buffer and repair these environmental pressures. Service ability emphasizes the enhancement of urban ecological service capacity through planning transformation so as to alleviate the overload operation of the ecosystem.

Institutional resilience refers to the guiding capacity of governments and non-governmental organizations to govern communities, the most important of which is the need to change political awareness and motivation so that cities can overcome obstacles under their guidance to achieve resilience and sustainability. Compared with other countries, China’s excellent performance in epidemic prevention and control is related to three factors. First, basic guarantee. In the face of the epidemic, the government eliminated the worries of patients by exempting treatment fees, thus avoiding the large-scale outbreak of anxiety among residents. Second, manpower input. After the outbreak of the epidemic, people from the national level to the local level have responded one after another. Strong grass-roots mobilization capacity provides effective guarantee for the realization of the full coverage of mass prevention and control or joint prevention and control. Third, financial input. In the early stage of the epidemic, the guiding principle of “proactive fiscal policy should be more proactive” is repeatedly emphasized, which provided a solid financial foundation and guarantee for epidemic prevention and control and stable economic operation.

Therefore, this paper selects corresponding indexes based on the above criteria layer, as Table 1 showing.

### 2.3. Data Sources

The time node of the data used in this paper is 2011–2018, and the original data of the basic indicators are from China City Statistical Yearbook and statistical yearbooks of provinces and cities, and part of the environmental index data is obtained from the bulletin of environmental quality of cities. Considering the availability of data, the research sample of this paper is 281 cities in China.

## 3. Results

### 3.1. Spatiotemporal Differentiation of China’s Urban Resilience

Identifying the spatial and temporal differentiation of urban resilience can effectively reveal its evolution process and potential formation mechanism [25], which is an important basis and premise for optimizing and improving urban resilience. Therefore, it is necessary to explore the temporal and spatial attributes of urban resilience. In order to effectively analyze spatiotemporal evolution of urban resilience, on the one hand, the urban resilience index of China from 2011 to 2018 is obtained by using the entropy method, and its evolution state in the time dimension is described in more detail by combining the α value and the global Moran’s I index. On the other hand, in order to observe the difference of urban resilience in spatial distribution more intuitively, ArcGIS10.3 software was used to draw the spatial pattern evolution map, and the hierarchical structure analysis and local Moran’s I were used to calculate and clarify the different development modes and spatial correlation of urban resilience in various regions.

### 3.2. Temporal Differentiation

#### 3.2.1. Overall Evaluation, Comprehensive Score and Subsystem Score

From the perspective of overall evaluation, the overall urban resilience index showed a continuous decline, from 0.0885 in 2011 to 0.0773 in 2018 (Table 2). The analysis shows that since 2011, China has gradually entered into the period of concentrated outbreak of “urban disease”. In the context of “emerging + transformation + rapid urbanization”, “acute, chronic, complications” in cities co-occur and resonate, triggering systemic risks of urban operation and resulting in continuous decline of urban resilience.

In terms of the sub-resilience, except the social resilience remained basically unchanged, the resilience of the remaining subsystems all decreased, among which the ecological resilience showed the most significant decline, with a decrease of 24.58% compared with 2011. The main reason is that China is in the critical stage of industrial development and transformation, and industries characterized by extensive, low-level and high energy consumption will be eliminated and transferred, which will have a significant impact on urban spatial layout and urban operation and management.

From the perspective of sequence transformation, economic resilience began to dominate, while engineering resilience decreased significantly. On the one hand, economic development plays an important role in urban resilience. The rapid economic development helps cities gather social wealth to improve their disaster response capacity. On the other hand, the engineering resilience dropped from the first place in 2011 to the third place. This may be due to the rapid expansion of population, the lack of predictability in urban planning, as well as the lag of infrastructure construction, which leads to the phased decline in the engineering resilience.

#### 3.2.2. Convergence Analysis

α convergence test is generally measured by the coefficient of variation, which can be used to directly reflect the changes in the distribution pattern of urban resilience in a region. For any t, there is αt + 1 < αt, indicating that the gap of urban resilience is gradually narrowing and there is α convergence [26].

On the whole (Table 2), the α value of China’s urban resilience is on the rise, which means that the α convergence does not occur, while the regional differences become more and more significant as time goes on. The α value of the sub-resilience is generally consistent with the overall resilience and shows an upward trend, while only the engineering resilience shows an obvious sign of convergence. This is because the outbreak of “urban disease” has accelerated the pace of infrastructure construction in various regions, thus gradually narrowing the gap of engineering resilience between regions.

**Figure 1:**
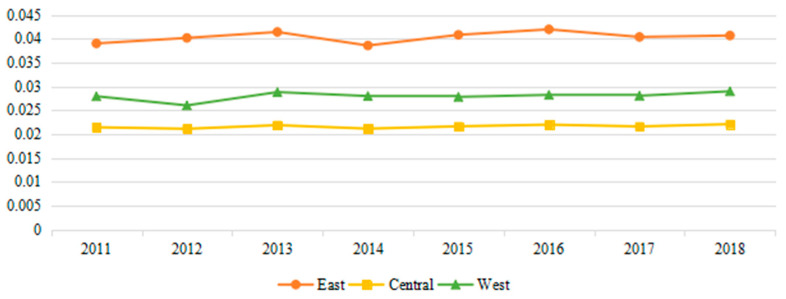
Trend chart of China’s urban resilience level from 2011 to 2018.

In terms of regions (Figure 1), urban resilience in eastern, central and western China shows no obvious signs of convergence. The reason lies in that the policy support at the national level has not yet met the overall urban development needs of each region, while the advantageous resources, driven by the tendency of profit and preference, are mostly concentrated in the relatively developed cities in each region. Therefore, the widening trend of differences between regional cities is presented.

#### 3.2.3. Global Moran’s I

The urban resilience of China is characterized by positive spatial autocorrelation, and Moran’s I value rises from 0.1717 in 2011 to 0.1812 in 2018 (Table 2). It indicates that the agglomeration trend in spatial distribution is gradually strengthening. However, in general, the overall Moran’s I index shows a trend of “decline first and then rise”, which may be due to the lack of strategic guidance in the overall planning of regional urban resilience development in the early stage, and the similarity degree of urban resilience in various regions decreases.

From the perspective of sub-resilience, economic resilience also has a trend of first decreasing and then rising, showing a significant positive correlation with the overall urban resilience. This once again proves that economic resilience has a great impact on urban resilience. The Moran’s I value of engineering resilience is low and presents an inverted “V” type, while the Moran’s I value of social resilience and ecological resilience are both higher than that of engineering resilience and present a positive “V” type. It indicates that the development of social resilience and ecological resilience will compress the improvement space of engineering resilience to some extent. However, the institutional resilience has been continuously declining, indicating that the agglomeration trend is weakening. This is mainly because cities rely too much on their own development needs and lack unified policy guidance in improving the resilience level, leading to an increasingly obvious regional trend of institutional resilience.

### 3.3. Spatial Differentiation

#### 3.3.1. Overall Spatial Pattern

**Figure 2:**
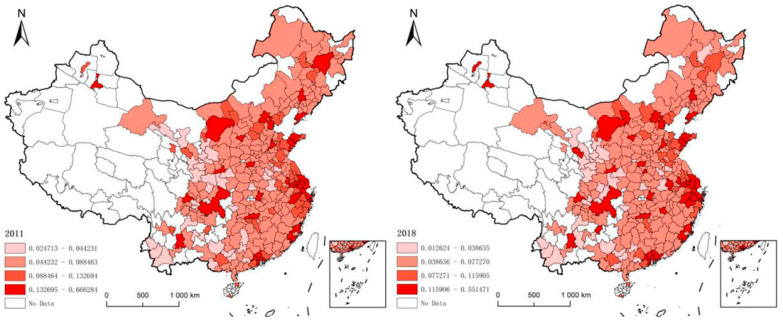
The spatial pattern evolution map of China’s urban resilience in 2011 and 2018.

In order to reflect the spatial pattern changes of urban resilience, this paper divides the resilience levels into low level, medium low level, medium high level and high level according to the regional economic classification standard of the World Bank and according to the mean values of resilience index of 50%, 100% and 150% (Figure 2) [27].

From the perspective of the quantity of each grade, there is no obvious change in the proportion from 2011 to 2018. In addition to the increase from 13% to 16% in the proportion of cities with high resilience, the proportion of cities with low and medium high resilience declined slightly, from 13% to 12%, and from 14% to 11%, respectively. Cities with medium low resilience levels have been dominant, with 169 in both 2011 and 2018. The main reason lies in the gradual exposure of low-end industrial structure in China, and the inability of ecological carrying capacity to adapt to the rapid development of urban economy, which leads to the low level of resilience.

From the perspective of spatial distribution, the spatial heterogeneity of urban resilience is obvious. The resilience level of eastern cities is generally higher than that of the central and western regions, and the high-value areas are steadily clustered in agglomerates in the Beijing-Tianjin-Hebei urban agglomeration, Shandong Peninsula and Yangtze River Delta urban agglomeration. This is because the eastern region relies on a reasonable industrial structure and perfect infrastructure to gather a large number of social resources, which ensures that the city can quickly respond to the disaster and bear the high cost of recovery. In contrast, urban resilience in the central and western regions is distributed in a “Mosaic” pattern. This is mainly reflected in the cluster agglomeration of cities with medium low-level resilience and point-like dispersion of cities with high level resilience. Moreover, there was no significant change in the point-like high-value areas in 2011 and 2018, which are dominated by provincial capitals and central cities in urban agglomerations, forming a certain spatial shielding effect [27]. Most of these cities produce strong siphon effect by virtue of policy advantages and constitute a self-consistent closed structural system. It does not have strong expansibility, and only through strong policy intervention can it enhance the radiation power.

#### 3.3.2. Hierarchical Structure Analysis

**Figure 3:**
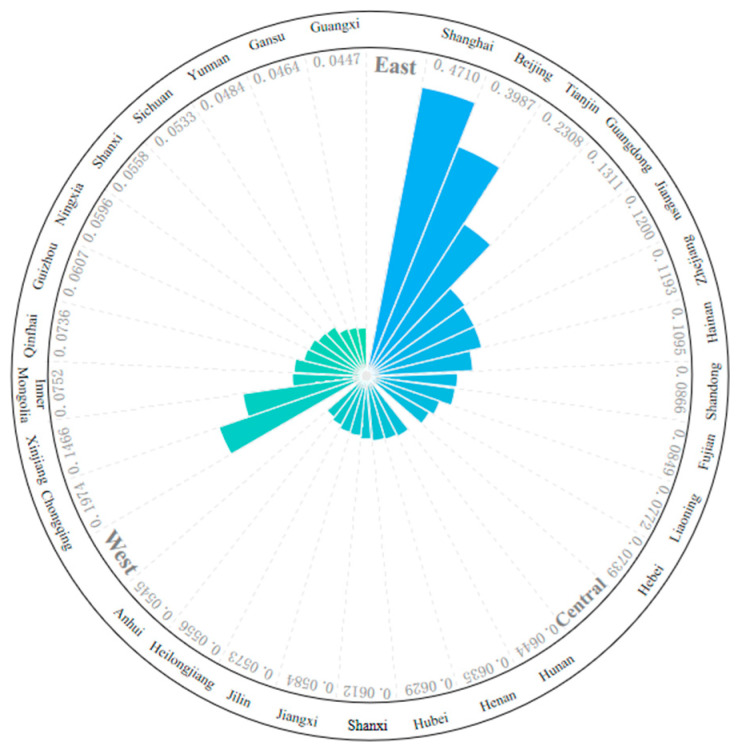
Urban resilience index of eastern, central and western provinces.

By analyzing the resilience levels of eastern, central and western China at the provincial level (Figure 3), the development model of urban resilience in various regions of China can be better understood. By observing the urban resilience index of various provinces in China, it is found that the resilience levels of the eastern and western provinces are greatly different, and there are significant polarization phenomena in both provinces. These high-value polarization areas are highly coincident with the distribution of Chinese municipalities directly under the central government, among which Shanghai is the most prominent. On the one hand, Shanghai has been making continuous efforts in urban planning, operation and management in recent years. On the other hand, it has increasingly strengthened its leading role as a central city through structural adjustment, innovative development and other measures, which has made a qualitative improvement of urban safety construction. In contrast, the level of development among the central provinces is very balanced, with no more prominent or backward provinces.

**Figure 4:**
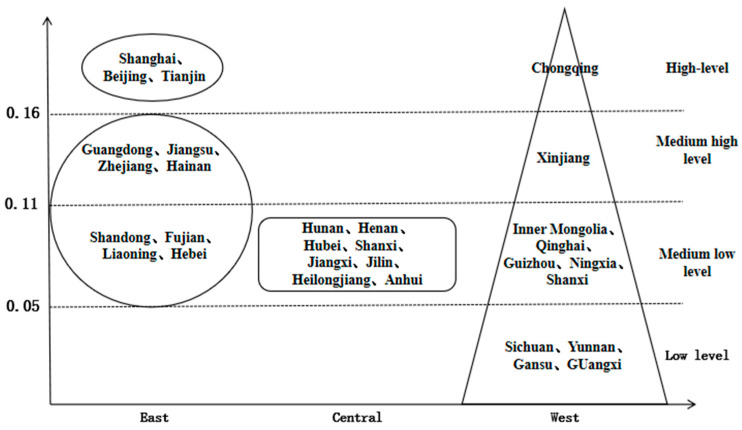
Urban resilience hierarchy in eastern, central and western China.

Combined with the ranking of urban resilience of each province, the three regions have different development patterns (Figure 4): (1) The eastern region has formed a three-core driving model supported by Beijing, Tianjin and Shanghai. Shanghai, Beijing and Tianjin, with their status as centers of politics, culture and international exchanges, have amassed resources and wealth, forming a relatively complete urban system. And with the gradual growth of the middle resilience class, it has some gradation, which enables the urban resilience of other provinces to ascend the ladder and alleviates the antagonistic emotions caused by the gap in resilience. (2) The central region is a rectangular structure. All the provinces in the region have medium low-level resilience, with the largest inter-provincial difference less than 0.01, lacking the “leader” with high resilience. This indicates that although the central region has a good development momentum in recent years, it cannot quickly catch up with the national average due to the original insufficient economic strength and insufficient supply of public goods and services, resulting in the low level of urban resilience. (3) The western region formed the pyramid structure. In this region, there are distinct layers and obvious polarization. Chongqing and Xinjiang have “fault exits” with high resilience levels of 0.1974 and 0.1466 respectively, while the rest of the provinces have gradually decreased. At the same time, the western region is also the only region with a low level of resilience, which undoubtedly exposes the imbalance of its urban resilience development. Due to the strong Matthew effect of Chongqing and Xinjiang themselves, the polarization of urban development in western China is extremely serious, which needs to be solved by the government to formulate more complete resource allocation policies in the future.

#### 3.3.3. Local Moran’s I

**Figure 5:**
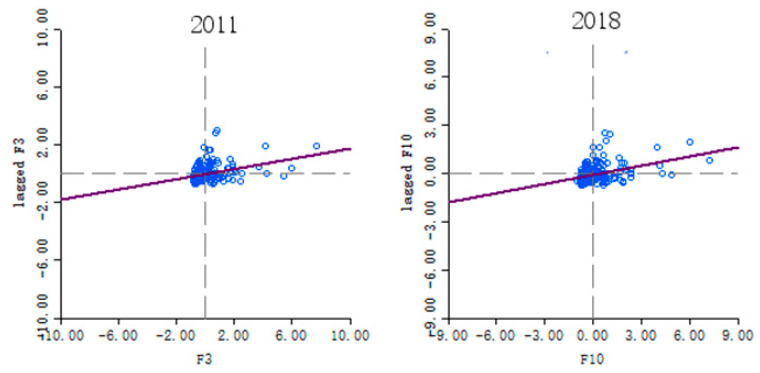
Local Moran’s I of urban resilience in China.

Local spatial autocorrelation analysis can effectively reveal the spatial characteristics of the correlation between local and surrounding urban resilience. The study found that the spatial characteristics of the city on the time cross section did not change substantially. And according to the adaptive cycle theory, this type of association was divided into four stages of urban development (Figure 5) [28]. (1) H-H type represents the city in the mature stage. This type of city is mainly concentrated in the developed regions dominated by the urban agglomeration of the Yangtze River Delta and Pearl River Delta. The economic leading position of these cities determines the high maturity of infrastructure and social development of these cities. In addition, in recent years, the pattern of coordinated development and the effect of environmental governance gradually appear, making the overall urban resilience higher in the region. (2) L-L type represents the city in the growth stage. A large part of them are located in city clusters in the Yellow River Basin. Different from cities in the Yangtze River Basin, cities along the Yellow River have not formed closely related economies, and many regions are resource-based cities, seriously restricting their economic, engineering and ecological resilience development. In addition, some cities are located in the southwest of China. The modern industry, which has been blank for a long time, makes the local economic structure, infrastructure construction and human quality backward. It is the “congenital deficiencies” that leads to the overall low urban resilience. (3) L-H type represents cities in the updating stage. There are only a few cities of this type. In 2011, Xuancheng and Langfang were included, while in 2018, Xuancheng was the only city left, and Langfang successfully changed into H-H type. It may be that the rapid development of Beijing-Tianjin-Hebei region has an increasingly strong radiation-driven effect on Langfang, making it more resilient. (4) H-L type represents the city in the gestation stage. It can be found that they all belong to the capital cities of western provinces, such as Kunming, Lanzhou and Chongqing. Compared with other cities, these cities can use the siphon effect to obtain more resources. However, there is not enough radiation effect to drive the development of small and medium-sized cities around them.

### 3.4. Spatial Scale and Spatial Differentiation of Influencing Factors

#### Index Construction

As an important realization path for sustainable development, urban resilience is not simply composed of a single subsystem but formed through the coordination and optimization of multiple elements, and the correlation strength between each element and the resilience index is different. Therefore, the enhancement of urban resilience must be based on a clear understanding of its impact mechanism. Referring to the research of existing scholars on influencing factors of urban resilience, this paper selects five indicators from government, technology, market, openness and financial factors to explore the mechanism of action of urban resilience (Table 3). Among them, the government factor played an important role in the battle against COVID-19, mainly through financial support. Changes in fiscal and tax policies often have the effect of “the slightest nudge causes the widest chain reaction” [29]. Moreover, the “real public needs”, with health care and education as the core, highlighted in the epidemic are difficult to be solved by market drive alone, and government public investment must be increased, so the infrastructure investment and financial level are used to represent its impact. The variables involved in technical factors include innovation input and human capital [30]. In Roosevelt’s New Deal period, the United States advocated “introducing the scientific spirit into the political and industrial fields”, which led to the peak of material environment construction in western developed countries. China began to lay out new infrastructure long before the epidemic, and this process must be supported by massive investment of innovation capital and human capital in the field of digital technology. Market factors focus on highlighting the domestic market capacity and market potential. The COVID-19 superimposed geopolitical disputes have challenged the external circulation model. However, with the domestic economy stepping to a new level, the market size and market potential are constantly expanding. All these have laid a foundation for the arrival of the great circulation era in China in the post-epidemic era [29]. Openness shows that the future city should actively respond to the call of domestic and international double circulation through foreign investment and foreign trade. The emphasis on “major domestic circulation” is not to close the door on the country, but to make the Chinese market more attractive to the world by expanding and strengthening the domestic market. Finally, “market exchange” is used to push forward globalization in the headwind, which is a process that forces cities to open up and reform. Financial factors include financial institutions scale and financial institutions efficiency. By expanding the financial institutions scale, social idle funds can be effectively gathered to support urban engineering construction, however, the acceleration of financial institutions efficiency will adversely affect urbanization and restrain the improvement of urban resilience [30].

### 3.5. Comparison of Models

Table 4 shows that the goodness of fit of MGWR is 0.948, which is significantly better than the GWR model. However, AICc value is far lower than the result of classical GWR model, indicating that it has a better measurement of urban resilience. On the one hand, the residual sum of squares of MGWR is smaller, indicating that it can obtain regression results closer to the true value with fewer parameters. On the other hand, MGWR reduces as much noise and bias in the regression coefficient as possible by allowing the existence of multiple action scales, thus enhancing its robustness. Therefore, the MGWR model is superior to the classical GWR model.

### 3.6. Scale Analysis

Different influencing factors have different heterogeneity and scale, that is, within a certain range, the effect size is similar, but beyond this range, the effect size is significantly different [20,31]. According to the action scales of different variables in MGWR and classical GWR (Table 5), GWR calculated the average value of each variable scale, so that all variables had the same action scale, with a bandwidth of 110, accounting for 39.1% of the total sample. In MGWR, differences are allowed in the action scale of all variables, and regression results show that all other variables are significant to different degrees except financial level. Specific manifestations are: (1) The bandwidth of innovation input, foreign trade and financial institutions scale are 87, 44 and 47 respectively, which are all micro-scale variables. The scale is close to the provincial level of China’s Xinjiang Autonomous Region. On the one hand, this indicates that they have a large spatial heterogeneity. Once the scale is exceeded, the coefficient will change dramatically. On the other hand, it also proves that urban resilience is very sensitive to these variables. (2) Market capacity, human capital, foreign investment and financial institutions efficiency are macro-scale variables, accounting for 44.8%, 39.9%, 42.7% and 52.7% of the total sample size respectively. This scale is close to the regional scale of northwest China, indicating that they are relatively stable in space. (3) The bandwidth of market potential and infrastructure investment reached 200 and 199 respectively, both of which are global scale variables. That is to say, they have little spatial heterogeneity.

### 3.7. Analysis of Coefficient Spatial Pattern

**Figure 6:**
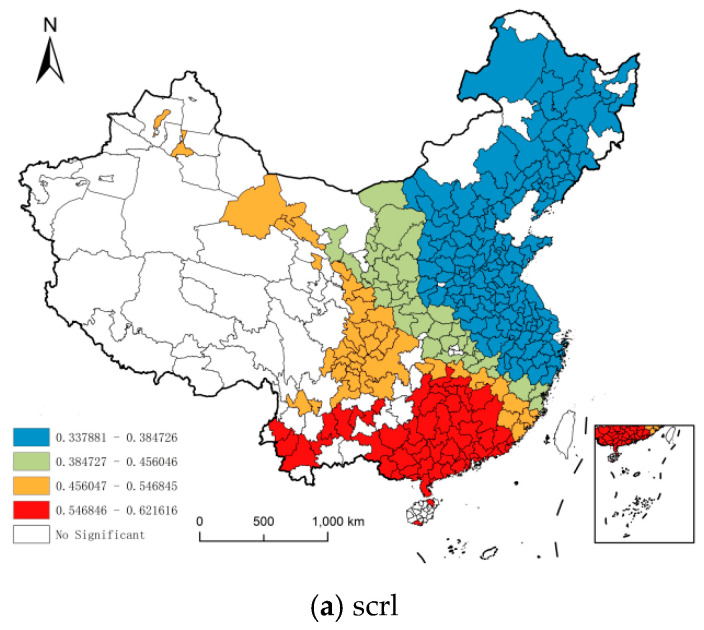

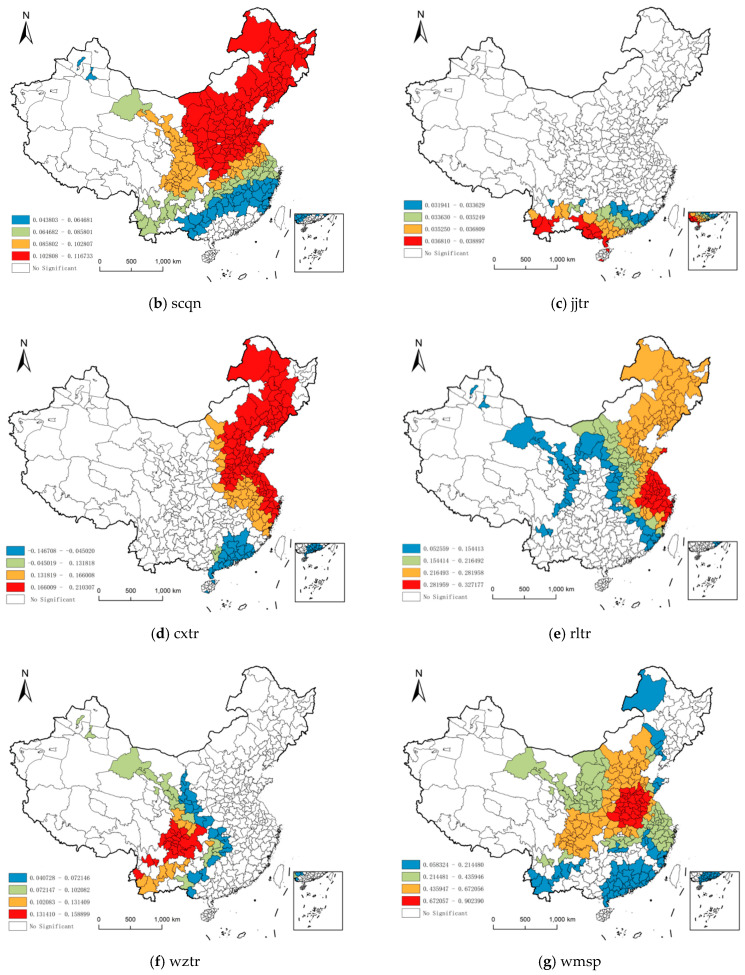

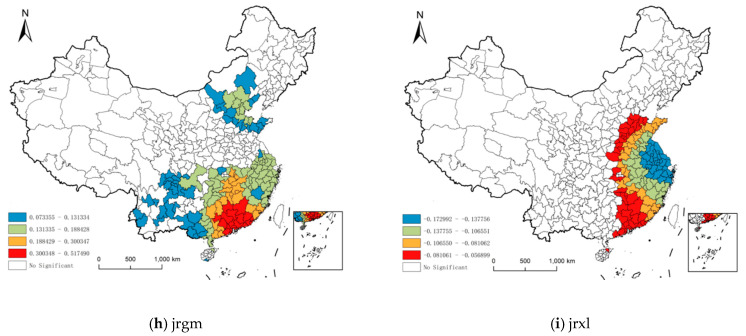
Spatial coefficient distribution of driving factors based on MGWR model.

The coefficient of market capacity is between 0.3379–0.6216, which has a significant positive effect on urban resilience, and the intensity is the highest among all variables, with an average value of 0.437. In the spatial layout (Figure 6a), there is an obvious polarization trend, with some cities in the southwest as the center of high-value polarization, while the low-value areas are mainly distributed in the central and eastern coastal areas. The high value areas include Guangxi, Hunan, Hainan, Jiangxi, Guizhou, Yunnan and Guangdong, which are all poor provinces except Guangdong. The poor in these areas have the strongest marginal propensity to consume and the lowest real consumption capacity, while the implementation of poverty alleviation and rural revitalization strategies directly drives investment in local agriculture and rural areas. On the one hand, by reducing the burden of medical care, health care, elderly care and education, we have accelerated the process of transforming rural residents’ economic income into production and living consumption, stimulated the regional consumer market, and injected strong momentum into urbanization. On the other hand, the expansion of market capacity directly stimulates the internal driving force of economic growth and strengthens the degree of marketization in a specific region. It will not only help improve the resilience of local cities, but also promote the resilience of surrounding areas through the radiation and diffusion of markets. The reason why low-value areas cluster in the economically developed central and eastern regions is probably because some cities try to secure long-term economic growth by acquiring market resources of neighboring cities. It not only weakens the market vitality of neighboring cities and makes them difficult to resist risks, but also causes the continuous decrease of spatial carrying capacity of their own systems and the prevention of external impact.

Market potential has a significant positive impact on urban resilience. The coefficient is between 0.0438 and 0.1167, with the mean value of 0.087 (Table 6). It indicates that 1% increase in population density will increase urban resilience by 0.087%, and the coefficient value indicates that its influence intensity is in the middle. There is an obvious step distribution trend in the space (Figure 6b), and some areas in south and east China are less affected by the market potential. The middle and lower reaches of the Yangtze River are the most prominent. Highly resilient cities in the region often contribute to urban development by gathering populations to replenish internal social capital and expand local markets. However, the increase of population density in specific cities will not only gradually “overdraw” the local environmental capacity, but also cause downward pressure on the engineering resilience of surrounding cities due to the outflow of resources. The areas most affected by the market potential are concentrated in the central and eastern areas dominated by the Yellow River Delta. Different from the above regions, the Yellow River Delta takes the ecological economic zone as an independent economic unit, devotes itself to the development of efficient ecological economy and insists on the synchronous improvement of productivity driven by scientific and technological innovation. By connecting a large number of major innovation platforms, projects, industries and talents, we can truly leverage high population concentration to increase productivity, thus stimulating the potential of cities and improving the social development momentum of neighboring cities.

The coefficient of infrastructure investment ranges from 0.0319 to 0.0389, and the impact on urban resilience is mainly concentrated in parts of southwest China and South China (Figure 6c). Although there is a significant positive impact, the intensity is the weakest. The mean coefficient and standard deviation are 0.016 and 0.018, indicating that the differences between regions are not significant. The spatial distribution shows a gradually decreasing trend from Yunnan and Guangxi to the northwest. The western regions have made full use of the development opportunities of urbanization and rural revitalization to get rid of the constraints of insufficient infrastructure. Based on the consideration of the public and external nature of infrastructure, the government has increased investment in the “real public needs” such as education, employment, medical treatment, social security and cultural life. It not only reduces the vulnerability and risk of local development, but also strengthens the urban hardware conditions and software environment, making it possible to carry out post-disaster restoration in a short time. In contrast, the Pearl River Delta region, as the vanguard of a new round of scientific and technological revolution and industrial transformation, the original “old infrastructure” can no longer meet the digital needs of traditional industries, only through the “new infrastructure” can we completely reshape the production relationship and release the digital productivity. The COVID-19 outbreak has also shown the world how digital technology can play a key role in shaping urban resilience. Different from “old infrastructure”, the investment in “new infrastructure” usually does not require the government to disclose the ins and outs. Instead, the market subject should guide the investment independently according to the policy and be responsible for the profits and losses. Therefore, the influence of government infrastructure investment on urban resilience is gradually weakened.

The coefficient of innovation input is between −0.1467 and 0.2103, and the local effect of innovation input on urban resilience is generally positive. This conclusion can be confirmed by the eastern coastal urban agglomerations dominated by Beijing-Tianjin-Hebei and Yangtze River Delta, but it has a negative spillover to the Pearl River Delta urban agglomeration (Figure 6d). As one of the three world-class urban agglomerations being cultivated in China, the negative effect of innovation input in the Pearl River Delta region is mainly reflected in the following points: (1) Lack of enough scientific and educational strength and innovation ability. The Pearl River Delta’s scientific and educational strength is inferior to that of the Beijing-Tianjin-Hebei region and the Yangtze River Delta in terms of the number of academicians of the Chinese Academy of Sciences and the number of the world’s top 500 universities. In addition, the technological development of the Pearl River Delta mainly relies on the introduction of advanced technologies, which leads to the lack of sufficient independent innovation ability and perfect innovation system, thus missing the opportunity to enhance urban resilience by promoting industrial structure optimization through innovation. (2) Competition for capital flow. On the one hand, the innovation of the Pearl River Delta is heavily dependent on the path of “self-sufficiency”, often through land finance to achieve “self-sufficiency”, which ultimately makes the new innovation city and new district become stale. On the other hand, the profit-driven capital makes enterprises more inclined to invest capital in real estate development after making profits, rather than innovation and research with a long return cycle. (3) Lack of collaborative innovation mechanism. Compared with the Beijing-Tianjin-Hebei collaborative innovation and the integrated development of the Yangtze River Delta, the essence of the negative spillover of innovation input in the Pearl River Delta lies in the liquidity of innovation resources. As the only urban agglomeration under the jurisdiction of just one province among the three, there are regional and institutional barriers among the innovation elements of the Pearl River Delta. For example, Although Shenzhen accounts for more than 50% of the patents in Guangdong, it is extremely short of basic innovation resources, while Guangzhou has nearly 70% of the universities and scientific research institutions in Guangdong, but it does not have enough innovation and transformation ability. Therefore, inefficient regional linkage further leads to the failure of technological progress to play a role in improving urban resilience.

The coefficient of human capital ranges from 0.0526 to 0.3272, which has a significant positive impact on urban resilience, with an average value of 0.165, indicating a strong impact intensity (Figure 6e). It is noteworthy that the contribution of labor factor input to China’s economic growth is more than 5 times that of capital factor input. It indicates that China’s dependence on capital factor in the process of economic growth is reduced, while the demand for labor factor input, especially the high level of human capital, is increased. In addition, the intensity of human capital’s influence on urban resilience is manifested strong in eastern region while weak in central China, which is consistent with the regional distribution of scientific and technological resources in China. The eastern cities in the Yangtze River Delta are trying to break down the barriers to build a “sharing model” for scientific research talents. By breaking through rigid constraints such as household registration and identity, the Yangtze River Delta is committed to establishing a flexible flow mechanism of talents between cities to accelerate the integration process of scientific research talents, so as to stimulate the potential high-quality labor force to contribute to the improvement of urban resilience. In contrast, provinces and cities in central China lack the policy advantages and economic basis of places like Beijing, Shanghai, Guangzhou and Shenzhen, and many talent development agreements are only stay on file, leading to the loss of a large number of scientific research talents and achievements. The lack of relevant supporting facilities for innovation activities, to a large extent, hinders the effective aggregation of self-owned knowledge and knowledge flow and inhibits the improvement of local innovation ability. At the same time, the strength to engage in basic research is obviously insufficient, the output of original scientific research achievements with world leading level is not high, and there are many shortcomings in science and technology that hinder the industrial transformation. This disconnect will make the industrial structure lag behind the economic development, which is not conducive to improving the resilience.

The coefficient of foreign investment ranges from 0.0407 to 0.1589, and the impact on urban resilience is only significant in the western region (Figure 6f). From the perspective of regression coefficient, the mean value is 0.04. Increasing foreign investment is conducive to improving the city’s ability to respond to the crisis. Based on the west development and the “One Belt and One Road” policy, the inland areas such as the northwest and southwest will no longer be restricted by the coastal radiation, but gradually become the frontier of the new opening up, which will greatly promote the agglomeration effect of foreign investment in the west. On the one hand, foreign investment to a large extent shows a positive spatial spillover effect. The increase of FDI in neighboring cities will promote that in the city, which will have a positive effect on the transfer of surplus agricultural labor force to urban non-agricultural industries. Meanwhile, foreign capital attracted by China is mainly concentrated in labor-intensive manufacturing industry, and the organic combination of labor-intensive FDI and rural surplus labor force will become a huge driving force for rapid economic development. On the other hand, foreign investment has positive influence on urban economic growth through technology spillover effect and regional innovation network effect. “One Belt and One Road” encourages foreign investment to gather in the western region in a large scale, thus accelerating the formation of the western innovation network. In addition, foreign-funded enterprises indirectly promote the technological progress and industrial upgrading of domestic enterprises through the spillover effect of technology, which makes the economy more and more diversity and thus enhances the vitality and resilience of urban economy. From the perspective of spatial distribution, Chengdu-Chongqing urban agglomeration has become the polarization center of western investment attraction. With “south channel” traffic location advantage, urban agglomeration of FDI with a strong “Matthew effect”, enhance its depth and breadth of opening to the outside world, FDI will no longer limited to the equipment field, but the whole industry chain layout, which can maximize the spillover effects of FDI, and promote urban resilience. 

Foreign trade level has a significant positive impact on the urban resilience. The coefficient is between 0.0583 and 0.9024, with an average of 0.347 (Figure 6g). Improving the level of foreign trade is conducive to the accumulation of resources and upgrading of industrial structure, thus forming the agglomeration effect. Urban agglomeration can not only improve the productivity of enterprises and cities, but also generate knowledge spillover effect to accelerate the accumulation of human capital so as to improve the innovation ability and R&D expenditure level of cities. Cities with high productivity and talent concentration tend to show stronger resilience in the face of external shocks. In addition, reducing the level of foreign trade between cities by expanding the openness can effectively stimulate the effect of foreign trade competition between neighboring cities, and also improve the urban resilience to a certain extent. The influence of foreign trade level has a certain circle structure, which is mainly concentrated in the Central Plains Urban Agglomeration and surrounding cities. The reason for this phenomenon may be that cities in central China are usually dominated by large manufacturing enterprises. This type of enterprise usually focuses on exporting one or several major products, but when faced with a sudden crisis such as serious epidemic, they tend to have more mobility and resource scheduling capability than coastal trading enterprises. At the same time, due to the lack of direct foreign ports, most central cities have special supervision areas with policy advantages, which have a stronger agglomeration effect on foreign trade activities and thus a stronger positive effect on urban resilience.

Financial institutions scale has a significant positive effect on urban resilience, and the coefficient is between 0.0736 and 0.5175, with an average value of 0.115 (Figure 6h). Expanding the scale of urban finance can effectively pool the social idle funds for industrial development and social construction, and its positive influence mechanism on urban resilience mainly includes three ways: (1) The appropriate expansion of financial institutions scale is conducive to enterprises for financing and improvement of technology to achieve rapid expansion of non-agricultural industries. Through the agglomeration of secondary and tertiary industries, the process of economic development and urbanization is continuously accelerated to improve urban resilience. (2) A larger financial institutions scale can effectively make up the gap between the supply and demand of urban public service facilities construction funds, by fully mobilizing all kinds of investors and operators to participate in the infrastructure construction to constantly strengthen urban engineering resilience. (3) The expansion of financial institutions scale helps to lower the minimum investment threshold of poor people in human capital, so that they can make human capital investment through financing to jump into the high-income groups. The high level of educational talents accumulated in this process can help the city to adjust the structure quickly and realize economic growth, and continuously improve the urban social resilience. The coefficient standard deviation of financial institutions scale is 0.114, which reflects that its influence on urban resilience varies greatly among different regions. Pearl River Delta is the most affected region. The reason may be that the Pearl River Delta, as the leading region of China’s economic reform and opening up, has a relatively good financial development foundation, and its superior geographical location makes economic extroversion obvious. At the same time, in recent years, the division of regional financial functions has been gradually clarified, and the dual-core features have become more and more prominent. These not only effectively promoted the Pearl River Delta financial integration process, but also strengthened its “siphon effect” on the financial scale, making the financial institutions scale present a significant “central-peripheral” structural feature in space.

The coefficient of financial institutions efficiency is between −0.1730 and −0.0569, which has a significant negative impact on urban resilience. However, the mean value of the coefficient is −0.057, indicating that the enhancement of financial institutions efficiency will reduce regional urban resilience to some extent (Figure 6j). The reasons for this phenomenon may lie in: From the perspective of short-term impact, financial institutions efficiency will restrain the development of employment urbanization in the short term. Because financial resources are too much in favor of urban economy, state-owned enterprises or large and medium-sized enterprises, they cannot effectively absorb surplus rural labor force and improve the urbanization rate, resulting in its adverse impact on urban society and economic resilience. In the long run, first of all, accelerating financial institutions efficiency may induce financial risks. The duality of urban economic development in China will lead to the inflow of capital into highly resilient urban enterprises under the guidance of policies, which will lead to unfair competition in financial resources between highly resilient and low-resilient cities, thus exacerbating financial risks and reducing urban resilience. Secondly, the development of financial institutions efficiency cannot provide long-term support for urban construction. Considering the risk-return of their own investment, most commercial financial institutions will not invest in urban construction, so the financial institutions efficiency will hardly have a significant impact on the resilience of urban engineering. However, the biggest negative impact of financial institutions efficiency is mainly concentrated in the Yangtze River Delta, which is probably due to the serious lag of financial integration in this region, leading to the divergence of financial institutions efficiency. The unbalanced distribution of financial resources makes the conversion of savings and investment also has great differences. For example, in recent years, the shortage of assets in Shanghai and the shortage of funds in Ningbo have further aggravated the regional financial risks and challenges and restricted the improvement of urban resilience.

## 4. Discussion

### 4.1. Conclusions

Based on the panel data of 281 cities in China from 2011 to 2018, the spatiotemporal differentiation characteristics of urban resilience in China are analyzed, and the driving mechanism of urban resilience is analyzed by using MGWR model. The results show that:

(1) From 2011 to 2018, the overall urban resilience and the sub-resilience have decreased to different degrees, and the sequence has changed significantly: economic resilience has gradually replaced the dominant position of engineering resilience. No significant α convergence occurred in China and some different regions, but it is noteworthy that the only convergence phenomenon occurred in the engineering resilience. In addition, the global Moran’s I show that the spatial agglomeration trend of China’s urban resilience has been continuously strengthened. In the subsystem, the spatial agglomeration degree of engineering resilience and the institutional resilience have decreased, while other subsystems have increased.
(2) In terms of spatial layout, cities with medium low-level resilience have always been in the core position. The distribution pattern of the eastern region being superior to the central and western regions has also been stable. Among them, some provinces in the eastern and western regions also show significant polarization. In addition, the development modes among the eastern, central and western regions are “three-core driving model”, “rectangular structure” and “pyramid structure” respectively. The Moran scatter plot explains the different development stages in different cities: The dominance of L-L (growth stage) cities has not changed. L-H (updating stage) and H-L (gestation stage) cities are mostly small and concentrated in the western region. H-H (mature stage) cities are mainly located in the Yangtze River Delta and Pearl River Delta.
(3) There are significant differences among scales of driving factors, reflecting that different variables have different levels of spatial heterogeneity. Among them, innovation input, foreign trade level and financial institutions scale are all micro-scale variables. Market capacity, human capital, foreign investment and financial institutions efficiency are macro-scale variables. Only market potential and infrastructure investment are global scale variables. In addition, most variables have a significant positive impact on urban resilience, while effect of innovation input and financial efficiency is different. And the order of influence intensity is: Market capacity > Foreign trade level > Human capital > Financial institutions scale > Market potential > Innovation input > Financial institutions efficiency > Foreign investment > Infrastructure investment. It can be further summarized as: Market factors > Opening factor > Technical factors > Financial factors > Government factor.

### 4.2. Suggestion

At present, the CoviD-19 epidemic is still out of control in the world. Although the prevention and control effect of the epidemic has been further shown, the spread trend is still very serious. From the perspective of the harm of the COVD-19 epidemic, the global spread and further deterioration of the epidemic will cause serious economic impact and social impact, which also indicates that China will be in the state of epidemic prevention and control for a long time. In order to meet the safe operation of Chinese cities under the background of normal epidemic, we put forward the following suggestions:

First, establish domestic circulation as the main to improve the market activity. From the analysis of influencing factors, it can be seen that market factors have the greatest impact on the development of urban resilience, so it is necessary to give full play to their role in improving urban resilience in the future. In addition, due to the multiple impacts of COVID-19 and trade protectionism, China’s consumer market has come to a new juncture of restructuring. Given that the world economy is in a state of global decoupling, China should stick to the domestic cycle as the main body and continue to expand domestic demand. On the one hand, we should release the purchasing power on the basis of improving social security system. (1) Perfect the market and the government combined with the flexible implementation mechanism, use of government administrative expenditure to strengthen the urban engineering resilience in poor areas. At the same time, with the help of the market force to improve the economy and social resilience, reduce the living burden of residents, stimulate consumption. (2) Actively develop online consumption, the spread of the epidemic ushered in the growth of various types of “residential economy”, so we must force enterprises to speed up the digital transformation, and strive to adapt to and promote the innovation of consumer mode. (3) For poor and backward areas, the rural cultural tourism industry can be built to strengthen the resilience of urban-rural relations, so as to realize the villagers’ nearby employment and increase farmers’ income, and fundamentally strengthening the power of domestic demand. On the other hand, we should rationally distribute urban population density and vigorously develop regional ecological economy. The central and eastern coastal areas should avoid the downward pressure of high population density on urban engineering and ecological resilience, and give full play to the positive role of public resource allocation in urban construction through unified market construction and rational population distribution, and strengthen green orientation in urban spatial planning. It also makes use of industrial upgrading, education promotion and innovation drive to enhance the endogenous development capacity of urban resilience.

Second, stick to open up and promote the domestic and international circulations. It can be seen from the above studies that openness plays an irreplaceable role in strengthening urban resilience development, so its influence on urban resilience development should be continuously exerted. By focusing on domestic economy, we will not shrink passively. Instead, we will counter the “anti-globalization” with high-level opening-up, counter the “divestment theory” by improving the business environment, and counter the “decoupling theory” by attraction of super-large markets, so as to speed up the formation of a more benign international economic cycle. First of all, we should actively seek new ideas for the development of foreign trade. Relying on the construction of free trade zones and free trade ports in various regions, we should speed up the cultivation of industrial clusters, bases or trading platforms with international influence, as well as high-quality business environment and sound market mechanism, and continuously expand the height, depth and breadth of opening-up by forming replicable and popularizable results. For exports, we need to make good use of the three major export outlets: domestic sales, cross-border e-commerce and import substitution. As for imports, we should take the initiative to reduce the tariff level so as to reduce the import cost of consumers, promote the balance of international payments, and enhance China’s voice in the world economy. Secondly, we should formulate differentiated opening-up policies. For the eastern cities, the primary task is to further expand the opening of logistics, research and development, digital economy and other service industries by relying on the construction of urban agglomerations, so as to accelerate the introduction of capital to help them quickly complete the “chain repair, chain expansion, chain strengthening” and form an efficient industrial chain and supply chain. We will build a world-class business environment as soon as possible, bring it into line with high-level international economic and trade rules, and gradually meet the requirements of legalization and facilitation of the business environment. For inland cities in the central and western regions, we should take “One Belt and One Road” as an important starting point and give full play to the advantages of the super-large market to speed up the construction of cooperative innovation networks. At the same time of relaxing the foreign exchange control of all kinds of capital in outbound acquisition, establish a cooperative innovation demonstration platform oriented to “One Belt and One Road”.

Third, break down the barrier of factor flow and improve urban “technical resilience”. The 21st century is the era of knowledge-based economy, and the fourth industrial revolution with intelligence as the core has just begun to appear. The role of technological factors in the resilient development of cities is self-evident, and the need to use technological innovation to shape a “new city” is increasingly urgent. From the perspective of economic resilience, the first step is to accelerate the formation of regional collaborative innovation networks conducive to the effective flow of innovative resources such as knowledge, technology, capital and equipment. The Pearl River Delta and other regions can start with strengthening top-level design, improve the strategic framework, support universities, laboratories, high-tech zones and other innovation carriers to form innovation alliances, and jointly push forward the joint tackling of key and core technologies. We should also give full play to the leading role of core cities. While complementing each other’s advantages, we should respect the regional agglomeration law of scientific and technological innovation, and jointly create regional innovation poles to accelerate the formation of an innovation space with global attraction and radiation power. At the same time, joint innovation fund will be set up to fund common regional science and technology and industrial projects, providing special funds for inter-provincial cooperation among enterprises, universities and research institutes. The second is to continuously promote the construction of talent integration, especially for the central region, the key lies in the formation of a balanced, complementary and dependent talent structure within the region. This not only requires all regions to coordinate their talent planning, train, allocate and guide the flow of talents in accordance with the principles of complementing each other’s advantages and strengthening their characteristics, but also actively explore a flexible mechanism for the flow of scientific and technological talents with permanent residence, no transfer of relationship, recognition of identities, and ability to leave and enter. In terms of engineering resilience, COVID-19 highlights the ability of “digital technology” to respond quickly to public health emergencies. In the future, the public health—geographic information data system can be built based on big data to warn potential risks of various urban public activities. In addition, regional intelligent governance can be carried out to continuously improve the scientific nature and high efficiency of regional resource allocation through data sharing and common information.

Fourth, we should improve the local financial development system and stimulate the endogenous driving forces for urban resilience. There is a close parallel relationship between urban economy and financial development, and in order to strengthen urban resilience, the healthy development of financial system must be realized. As an important factor in promoting urban resilience development, the role of financial factors still needs to be further strengthened. From the perspective of the financial system itself, the key lies in the realization of regional financial integration. Specific paths include: (1) Improving the financial resources allocation system. Firstly, it is necessary to establish a multi-level and wide-ranging financial market system to create favorable market conditions for regional financial cooperation and innovation. Secondly, regional financial infrastructure system should be improved, including trans-regional enterprise credit investigation service platform, electronic payment and settlement platform and financial information sharing system. (2) Jointly building a financial innovation service system. Its focus is to build a technology and financial service system to support the transformation of scientific and technological achievements and the industrialization of scientific and technological projects. It is also necessary to cultivate leading cities of fintech and standard innovation as soon as possible and promote the agglomeration of fintech talents, technologies and capital to the region. (3) Create a characteristic financial industry system. Set up pilot zones for financial reform and demonstration zones for characteristic financial industries around modern industries, and gradually form a characteristic financial service system of “finance + modern industry”. In order to promote urbanization, the role of finance in social, economic and engineering resilience should be emphasized. (1) To guide more financial resources to the field of education, to meet the floating population children’s education and vocational training needs, so as to transform the dividend of population resources into human capital dividend. (2) Control financial resources do not excessively concentrate on state-owned enterprises, large and medium-sized enterprises, and for small and medium-sized enterprises or industrial clusters “share soup”, so as to effectively promote the employment of rural surplus labor force. (3) Through providing financial support for high-quality agricultural products and agricultural production technology to promote agricultural industrialization and rural industrialization development. (4) Establish a diversified and market-oriented urban construction financing system to raise funds for urban infrastructure construction.

Sixth, strengthen government functions and release the institutional resilience. As a new field of urban public management, resilient urban construction has not fully exerted its institutional resilience. Therefore, it is necessary to establish a decision-making and coordination mechanism dominated by the government and involving multiple departments and subjects. Firstly, improve the assessment classification system. It includes two parts: classification assessment index and emergency treatment index. On the one hand, we should divide the urban resilience into different risk levels and development stages to construct scientific assessment indexes, so that all cities can “fit into each other”. On the other hand, the emergency treatment indicators should be improved to provide differentiated policy support for different types of cities. Among them, developed cities should focus on experience exploration and innovation, while high-risk cities should avoid increasing social and economic vulnerability or disaster amplification effect in the rapid urbanization process. Secondly, construct public security system. The system should not only include the four subsystems of early warning system, prevention system, emergency response system and reconstruction system, but also include four levels of prevention and control: national—provincial—city—county—township (community). Thus, it forms a network mode with national macro control, provincial regional coordination, perfect city and county layout facilities and township (community) support. Thirdly, combine political responsibility and legal responsibility. In the whole process of undertaking resilient urban construction, the government should run through the legal warning line, and formulate legal basis for disaster response, especially for disaster prevention and mitigation at the community level, so as to effectively define the responsibilities of governments at all levels. Fourthly, improve the infrastructure resilience as a guarantee. The shortage of medical facilities is the biggest pain point of the COVID-19 outbreak. This reminds us that while improving the infrastructure, we should not only consider the normal functional requirements, but also consider the various requirements in case of unexpected risks. Pay attention to the construction and use of mobile facilities and equipment, and the construction of lifeline projects, so as to increase the redundancy of urban shock resistance.

## Figures and Tables

**Table 1:** Urban resilience evaluation index system.

Target Layer	Rule Layer	Factor Layer	Index Layer
Urban resilience	Economic resilience	Economic strength	GDP per capita
			Per capita savings balance
			Industrial enterprises above designated size
		Economic diversity	Ratio of output value of secondary and tertiary industries to GDP
		Economic extroversion	The number of foreign-invested enterprises in industrial enterprises above designated size
			Foreign direct investment contract projects
	Engineering resilience	urban evacuation ability	Road area per capita
			The number of buses per 10,000 people
		Basic supply and drainage ability	Drainage pipe density
			Per capita electricity consumption of city
			Total urban gas supply
			Water consumption per capita
		External communication ability	Number of households with Internet broadband
			Number of mobile phone users at the end of year
	Social resilience	Population adaptation ability	Natural population growth rate
			Urban unemployment rate
		Social security ability	Social security Index
			Number of beds per 10,000 persons
		Risk response ability	The number of college students per 10,000 persons
			Health technicians per 10,000 persons
	Ecological resilience	Environmental pressure	Industrial wastewater discharge per unit of GDP
			Industrial smoke (dust) emissions per unit of GDP
		Governance ability	Comprehensive utilization rate of general industrial solid waste
			Harmless disposal rate of household garbage
		Service ability	Green coverage in built-up areas
			Park green area per capita
	Institutional resilience	Basic guarantee	Unemployment insurance coverage
			The number of urban workers participating in basic medical insurance
		Manpower input	Proportion of employees in public administration and social organizations
		Financial input	Fiscal spending

**Table 2:** Changes of urban resilience index, α value and Moran’s I index in China.

Urban Resilience System	Resilience Index			α Value			Moran’s I		
	2011	2015	2018	2011	2015	2018	2011	2015	2018
Overall resilience	0.0885	0.0837	0.0773	0.0297	0.0303	0.0308	0.1717	0.1679	0.1812
Economic resilience	0.0211	0.0202	0.0204	0.0297	0.0303	0.0308	0.2001	0.1931	0.2272
Engineering resilience	0.0237	0.0208	0.0189	0.0329	0.0309	0.0294	0.0508	0.0536	0.0447
Social resilience	0.0197	0.0210	0.0198	0.0295	0.0312	0.0344	0.0667	0.0657	0.0725
Ecological resilience	0.0118	0.0101	0.0089	0.0088	0.0086	0.0098	0.1345	0.0715	0.1499
Institutional resilience	0.0122	0.0115	0.0093	0.0332	0.0383	0.0396	0.1265	0.1061	0.1048

**Table 3:** Indicators of driving forces of urban resilience.

Factors	Indicators	Measurement
Government factor	Infrastructure investment	Investment in the construction of municipal public facilities/Total fixed asset investment
	Financial level	Fiscal revenue/GDP
Technical factors	Innovation input	Technology expenditure/GDP
	Human capital	Number of R&D employees per 10,000 persons
Market factors	Market capacity	Total retail sales of social consumption per capita
	Market potential	The population density
Opening factors	Foreign investment	FDI/GDP
	Foreign trade level	Total import and export/GDP
Financial factors	Financial institutions scale	The total amount of deposits and loans of financial institutions/GDP
	Financial institutions efficiency	Financial loan-to-deposit ratio

**Table 4:** Comparison of GWR and MGWR model indicators.

Model Indexes	MGWR	GWR
R2	0.948	0.937
AICc	135.171	182.703
Sum of squares of residuals	14.715	17.828

**Table 5:** Bandwidth of GWR and MGWR models.

Variable	MGWR	GWR
Market capacity	126	110
Market potentia	200	110
Infrastructure investment	199	110
Financial level	276	110
Innovation input	87	110
Human capital	112	110
Foreign investment	120	110
Foreign trade level	44	110
Financial institutions scale	47	110
Financial institutions efficiency	148	110

**Table 6:** Statistical description of MGWR regression coefficient.

Variable	Definition	Mean	Standard Deviation	Min	Median	Max
scrl	Market capacity	0.437	0.101	0.338	0.39	0.622
scqn	Market potentia	0.087	0.027	0.032	0.098	0.117
jjtr	Infrastructure investment	0.016	0.018	−0.147	0.021	0.039
cxtr	Innovation input	0.065	0.093	−0.147	0.089	0.21
rlzb	Human capital	0.165	0.096	0.018	0.156	0.327
wztr	Foreign investment	0.04	0.045	−0.029	0.024	0.159
wmsp	Foreign trade level	0.347	0.248	−0.043	0.314	0.902
jrgm	Financial institutions scale	0.115	0.114	−0.097	0.098	0.517
jrxl	Financial institutions efficiency	−0.057	0.052	−0.173	−0.052	0.023

## Data Availability

The data presented in this study are openly available in National Bureau of Statistics, reference number 978-7-5037-9120-8, 978-7-5037-8770-6, 978-7-5037-8432-3, 978-7-5037-8082-0, 978-7-5037-7706-6, 978-7-5037-7350-1, 978-7-5037-7019-7, 978-7-5037-6754-8.
